# Selective Targeting of Non-nuclear Estrogen Receptors with PaPE-1 as a New Treatment Strategy for Alzheimer’s Disease

**DOI:** 10.1007/s12640-020-00289-8

**Published:** 2020-10-06

**Authors:** Agnieszka Wnuk, Karolina Przepiórska, Joanna Rzemieniec, Bernadeta Pietrzak, Małgorzata Kajta

**Affiliations:** Department of Experimental Neuroendocrinology, Laboratory of Molecular Neuroendocrinology, Maj Institute of Pharmacology, Polish Academy of Sciences, Smętna street 12, 31-343 Krakow, Poland

**Keywords:** Alzheimer’s disease, Neurodegeneration, Neuroprotection, Estrogen receptors, Non-nuclear ERs

## Abstract

Alzheimer’s disease (AD) is a multifactorial and severe neurodegenerative disorder characterized by progressive memory decline, the presence of Aβ plaques and tau tangles, brain atrophy, and neuronal loss. Available therapies provide moderate symptomatic relief but do not alter disease progression. This study demonstrated that PaPE-1, which has been designed to selectively activate non-nuclear estrogen receptors (ERs), has anti-AD capacity, as evidenced in a cellular model of the disease. In this model, the treatment of mouse neocortical neurons with Aβ (5 and 10 μM) induced apoptosis (loss of mitochondrial membrane potential, activation of caspase-3, induction of apoptosis-related genes and proteins) accompanied by increases in levels of reactive oxygen species (ROS) and lactate dehydrogenase (LDH) as well as reduced cell viability. Following 24 h of exposure, PaPE-1 inhibited Aβ-evoked effects, as shown by reduced parameters of neurotoxicity, oxidative stress, and apoptosis. Because PaPE-1 downregulated Aβ-induced *Fas*/FAS expression but upregulated that of Aβ-induced *FasL*, the role of PaPE-1 in controlling the external apoptotic pathway is controversial. However, PaPE-1 normalized Aβ-induced loss of mitochondrial membrane potential and restored the BAX/BCL2 ratio, suggesting that the anti-AD capacity of PaPE-1 particularly relies on inhibition of the mitochondrial apoptotic pathway. These data provide new evidence for an anti-AD strategy that utilizes the selective targeting of non-nuclear ERs with PaPE-1.

## Introduction

Alzheimer’s disease (AD) is a multifactorial and severe neurodegenerative disorder characterized by progressive memory decline, the presence of Aβ plaques and tau tangles, brain atrophy, and neuronal loss. The sporadic form of AD has a late onset and accounts for over 95% of all cases. Aβ has a pivotal role in the pathogenesis of AD, and insoluble clusters or intermediary soluble oligomers of Aβ have been implicated in neurotoxicity and cell death. Aβ peptides are produced by proteolytic cleavage of APP (Nicolas and Hassan 2014). In the amyloidogenic pathway, APP is initially cleaved by β-secretase to produce a soluble secreted form of amyloid precursor protein (APP) and a fragment βAPP-CTF; subsequent cleavage of βAPP-CTF by γ-secretase yields the Aβ peptide and amyloid precursor protein intracellular domain (AICD). Because γ-secretase can cleave at several alternative sites, the resulting Aβ peptides vary in length. The most abundant forms found in amyloid plaque are the 40-mer and the 42-mer (De Strooper 2010). However, there are inconsistencies and controversies surrounding the amyloid hypothesis of AD (Morris et al. 2014). Recently, an age-dependent hypothesis of AD has been proposed that integrates the old amyloid cascade hypothesis as part of the pathological progression. A report from the World Health Organization (WHO) and Alzheimer’s Disease International (ADI) calls for governments and policymakers to make dementia a global public health priority because approximately 44 million people worldwide have the disease, a number that is predicted to triple by 2050. Currently, there is no cure to stop the progression of Alzheimer’s disease. With an estimated global cost of over $600 billion, new therapeutic approaches are urgently needed. As available therapies provide moderate symptomatic relief but do not alter disease progression, novel therapies are needed.

AD is accompanied by dysregulation of estrogen receptor (ER) signaling, including non-nuclear ER signaling. Transcriptome meta-analysis has revealed a central role for sex steroids in the degeneration of neurons in AD (Winkler and Fox 2013). Indeed, risk for AD is associated with age-related loss of sex steroid hormones in both women and men. In postmenopausal women, the precipitous depletion of estrogens and progestogens is hypothesized to increase susceptibility to AD pathogenesis. Similarly, age-related testosterone loss is associated with an increased risk of the disease in men (Pike et al. 2009). Despite similarities, the incidence of dementia and AD is at least twofold higher in women than in men. Several studies have suggested that decreased adult neurogenesis plays a role in the initiation and progression of neuropathology in AD. Furthermore, the combined effect of slightly elevated Aβ levels and oxidative stress due to aging has been proposed to initiate AD long before clinical onset (Stockburger et al. 2014).

Emerging evidence suggests that there is a “critical period” for estradiol’s beneficial effect in the brain. The critical window hypothesis suggests that hormone therapy initiated at a younger age in closer temporal proximity to menopause may reduce the risk of AD (Scott et al. 2012). However, the application of estrogens as neuroprotectants in humans presents numerous limitations, including adverse effects on peripheral tissues. In addition to classical nuclear ERα (ESR1) and ERβ (ESR2) acting as ligand-activated transcription factors, it has become evident that non-nuclear ERs govern numerous cell processes in the brain and exert beneficial cardiometabolic effects without uterine or breast cancer growth in mammals. Non-nuclear ERs are localized on cell membranes and include mERα, mERβ, GPR30 (GPER1), and Gq-mER. Activators of non-nuclear ERs share neuroprotection attributed to estradiol and phytoestrogens, but there is no report on their involvement in anti-AD therapy.

PaPE-1 ((S)-5-(4-hydroxy-3,5-dimethyl-phenyl)-indan-1-ol) is a “pathway preferential estrogen” that interacts with the extranuclear ER signaling pathway without activating the nuclear signaling pathway (50,000 less bound to nuclear receptors). The mechanism of action of PaPE-1 does not result in negative effects on the reproductive system or breast cancer cell proliferation (Madak-Erdogan et al. 2016). PaPE-1 does not induce ERα or ERK2 recruitment to gene enhancers or stimulate expression of proliferation-associated genes, as seen with E2. However, similar to E2, PaPE-1 strongly activates the MAPK and mTOR pathways, and as based on the effects of MAPK and mTOR inhibitors, PaPE-1 relies on these pathways for a considerable proportion of its gene regulation. In non-reproductive tissues, PaPE-1 has been demonstrated to repair the vascular endothelium after injury and reduce adipose stores and blood triglyceride concentrations. Moreover, PaPE-1 decreased stroke severity, attenuated neuroinflammation, and promoted functional recovery in mice without undesirable uterotrophic effects (Selvaraj et al. 2018). Regarding the neuroprotective capacity of membrane estrogen receptors, it has recently been shown that activation of GPR30 ameliorates memory impairment in a mouse model of AD (Kubota et al. 2016) and protects against Aβ toxicity in vitro (Gray et al. 2016; Deng et al. 2017).

Since PaPE-1 has the ability to selectively activate non-nuclear ERs without evoking adverse hormonal effects, we aimed to assess the neuroprotective properties of it in a cellular model of sporadic AD. We hypothesized that targeting non-nuclear ERs with PaPE-1 will prevent Aβ-induced toxicity in mouse brain neurons in primary culture.

## Materials and Methods

### Primary Neuronal Cell Culture

Primary neocortical cultures were prepared from E15 embryos (CD-1® IGS Swiss mouse, Charles River, Germany) as previously described (Wnuk et al. 2020). Embryonic cortices were minced into small pieces and incubated with 0.1% trypsin for 15 min at 37 °C. The cells were placed in medium containing 10% fetal bovine serum (Sigma-Aldrich, USA) and centrifuged for 5 min at 1500×*g*. The neuronal cells were seeded on poly-L-ornithine-coated (0.1 mg per ml; Sigma-Aldrich, USA) plates at a density of 2.0 × 10^5^ cells per cm^2^ in multiwell plates (TPP Techno Plastic Products AG, Switzerland) and cultured in neurobasal medium (Thermo Fisher Scientific, USA) containing L-glutamine (Sigma-Aldrich, USA), B27 (Thermo Fisher Scientific, USA) and penicillin-streptomycin antibiotics (Sigma-Aldrich, USA) at 37 °C in a humidified atmosphere containing 5% CO_2_ for 7 days in vitro (DIV).

All animals used in the research were maintained according to the principles of the Three Rs in compliance with European Union Legislation (Directive 2010/63/EU, amended by Regulation (EU) 2019/1010).

### Treatments

Aβ 1-42 (rPeptide, USA) was prepared as previously described (Messori et al. 2013). Briefly, aggregates of Aβ 1-42 were eliminated with HFIP (hexafluoroisopropanol, Sigma-Aldrich, USA). Next, HFIP was removed under N_2_ flux, and Aβ was dissolved in DMSO (Sigma-Aldrich, USA). Primary neocortical cell cultures were treated with 5–20 μM Aβ for 6 and 24 h. The neuroprotective effect against Aβ was examined with the use of PaPE-1 ((S)-5-(4-hydroxy-3,5-dimethyl-phenyl)-indan-1-ol, 0.01–10 μM) purchased from Sigma-Aldrich, USA. All compounds were dissolved in DMSO (dimethyl sulfoxide, Sigma-Aldrich, USA), not exceeding a concentration of 0.1% in the culture medium.

### Measurement of Lactate Dehydrogenase Release

Lactate dehydrogenase (LDH) release was measured in the cell culture supernatant at 6 and 24 h after Aβ and/or PaPE-1 administration with the use of a Cytotoxicity Detection Kit (Roche, Switzerland) as previously described (Wnuk et al. 2020) and according to the manufacturer’s protocol. The intensity of the red color (formazan salt) was measured at 490 nm and determined as a proportion of the LDH activity. The measurements were performed using an Infinite M200PRO microplate reader (Tecan, Switzerland), and the results were analyzed by i-control software. The data were normalized to the blank, and the results are presented as a percentage of the control ± SEM.

### Assessment of Caspase-3 Activity

Caspase-3 activity was assessed as described previously (Wnuk et al. 2020). Briefly, cells were lysed with lysis buffer containing DTT (DL-dithiothreitol, Sigma-Aldrich, USA) and incubated with caspase-3 colorimetric substrate—Ac-DEVD-*p*NA (N-acetyl-asp-glu-val-asp-*p*-nitroanilide; Sigma-Aldrich, USA) at 37 °C. Levels of the caspase-3 reaction product (*p*-nitroanilide) were measured for 60 min at 405 nm with an Infinite M200PRO microplate reader (Tecan, Switzerland). The results were analyzed by using i-control software and normalized to the absorbance of vehicle-treated cells. The data are presented as a percentage of the control ± SEM.

### Assessment of the Mitochondrial Membrane Potential

Mitochondrial membrane potential was measured with JC-1 Assay Kit (Biotium Inc., USA) as previously described (Rzemieniec et al. 2018; Wnuk et al. 2018a; Wnuk et al. 2018c; Kajta et al. 2019). According to the manufacturer’s protocol, aggregation of the JC-1 dye occurs in healthy cells with intact mitochondrial membranes, with intense red fluorescence. In cells with low mitochondrial membrane potential, the JC-1 dye remains in the cytoplasm in a green fluorescent monomeric form. Red (550 nm/600 nm) and green (485 nm/535 nm) fluorescence intensities were measured using an Infinite M200PRO microplate reader (Tecan, Switzerland). The data were analyzed using Tecan i-control software and normalized to the fluorescence intensity of vehicle-treated cells; the results are expressed as the red to green fluorescence ratio.

### Measurement of ROS Activity

Reactive oxygen species (ROS) activity was measured with the use of H_2_DCFDA (2′,7′-dichlorodihydrofluorescein diacetate, 5 μM) as previously described (Wnuk et al. 2018a). H_2_DCFDA is cell permeable and deacetylated by cellular esterases to produce H_2_DCF (2′,7′-dichlorodihydrofluorescein), which is rapidly oxidized by ROS to highly fluorescent 2,7′-dichlorofluorescein (DCF). DCF fluorescence was measured with excitation and emission wavelengths of 498 and 522 nm, respectively, using an Infinite M200PRO microplate reader (Tecan, Switzerland). The data were analyzed using Tecan i-control software and normalized to the fluorescence intensity of vehicle-treated cells (% of control).

### Assessment of Cell Viability

For monitoring cell viability, CellTiter-Blue® Cell Viability Assay (Promega, USA) was applied according to the manufacturer’s protocol. The assay is based on the reduction of resazurin to resorufin and is proportional to the number of viable cells. Cells were incubated with CellTiter-Blue® Reagent for 1 h, and then fluorescence was measured at 560/590 nm using an Infinite M200PRO microplate reader (Tecan, Switzerland). The data were analyzed using Tecan i-control software and normalized to the fluorescence intensity of vehicle-treated cells (% of control).

### qPCR Analysis of *Fas*, *FasL*, *Bax*, *Bcl2*, and *Gsk3b* mRNA

Total RNA was extracted from neocortical cell cultures at 7 DIV with reagents from an RNeasy Mini Kit (Qiagen, USA) according to the manufacturer’s protocol, as previously described (Wnuk et al. 2018b; Wnuk et al. 2019; Wnuk et al. 2020). The RNA was eluted in 40 μl of RNAse-free water. The amount of RNA was spectrophotometrically determined at 260 nm, and a 260/280 nm ratio was obtained (ND/1000 UV/Vis; Thermo Fisher, NanoDrop, USA). An A260/A280 ratio of ~ 2.0 is accepted as indicative of pure RNA. The RNA extract was reverse transcribed immediately after isolation to avoid freeze-thaw cycles. The RNA quality (integrity) was analyzed using PrimePCR™ RNA Quality Probe Assay, Mouse (Bio-Rad, USA). Total RNA was reverse transcribed with a High-Capacity cDNA Reverse Transcription Kit (Thermo Fisher Scientific, USA) according to the manufacturer’s protocol with a T100 Thermal Cycler (Bio-Rad, USA). The collected cDNA was stored overnight at − 20 °C and used for quantitative polymerase chain reaction (qPCR) on the next day. The cDNA was amplified using FastStart Universal Probe Master (Roche, Switzerland) containing TaqMan Gene Expression Assays (Thermo Fisher Scientific, USA) specific for *Fas*, *FasL*, *Bax*, *Bcl2*, and *Gsk3b*. For amplification, a mixture containing 10 μl of FastStart Universal Probe Master, 1 μl of cDNA as template, 1 μl of the TaqMan Gene Expression Assay mix, and 8 μl of RNase-free water in a total volume of 20 μl was used. The qPCR procedure using a CFX96 Real-Time system (Bio-Rad, USA) was performed as follows: 2 min at 50 °C and 10 min at 95 °C, followed by 40 cycles of 15 s at 95 °C and 1 min at 60 °C. The data were analyzed using the delta Ct method. The reference gene was chosen with the use of the following algorithms: geNorm, NormFinder, BestKeeper, and delta Ct; the 3 algorithms recommended glyceraldehyde-3-phosphate dehydrogenase (*Gapdh*) as the most stable reference gene.

### ELISAs of FAS, BAX, and BCL2

Protein expression of FAS, BAX, and BCL2 in neocortical cells at 24 h after exposure to Aβ was determined using enzyme-linked immunosorbent mouse-specific assays (ELISAs; Bioassay Technology Laboratory, China) according to the manufacturer’s protocol and as described previously (Wnuk et al. 2020). Absorbance was measured at 450 nm, and the values were correlated with the amounts of the specific proteins. The total concentrations of the proteins in the samples were estimated with a Bio-Rad protein assay based on the method of Bradford (Bio-Rad, USA) using bovine serum albumin (Sigma-Aldrich, USA) as a standard. The level of each protein measured is expressed as a percentage of the control ± SEM.

### Western Blot Analysis

After experiment, the neocortical cells were lysed in RIPA lysis buffer with protease inhibitor. The solution was sonicated and centrifuged at 15,000×*g* for 20 min at 4 °C. To determine protein concentration, Bradford reagent (Bio-Rad Protein Assay, USA) and bovine serum albumin (as a standard) were used. Samples that contained 35 μg of total protein reconstituted and denaturated in the Laemmli sample buffer and next the proteins were separated using 7.5% SDS-polyacrylamide gel (Bio-Rad, USA). After electrophoresis, the proteins were electrotransferred from gel to the PVDF membranes using the Bio-Rad Mini Trans-Blot apparatus. To block the non-specific binding sites, the membranes were washed with 5% dried milk and 0.2% Tween-20 in 0.02 M TBS (Tris-buffered saline) for 2 h. During the night, the incubation of membranes at 4 °C with one of the chosen primary antibody (Santa Cruz Biotechnology, USA) diluted in TBS/Tween: anti-β-Actin mouse monoclonal antibody (diluted 1:3500), anti-BAX rabbit polyclonal antibody (diluted 1:100), anti-BCL2 rabbit polyclonal antibody (diluted 1:100), and anti-FAS rabbit polyclonal antibody (diluted 1:100) occurred. Following, the membranes were washed and incubated for 2 h with horseradish peroxidase-conjugated IgG in TBS/Tween 20 (diluted 1:1000). The chemiluminescent signal was detected using BM Chemiluminescence Blotting Substrate (Roche Diagnostics GmBH) and visualized with a Luminescent Image Analyzer Fuji-Las 4000 (Fuji, Japan). Immunoreactive bands were quantified using MultiGauge V3.0 (ScienceLab).

### Statistical Analysis of the Data

Statistical tests were performed on raw data. The results are expressed as the mean absorbance or fluorescence intensity (in arbitrary units) per well containing 50,000 cells for analyses of caspase-3 activity and LDH release and as fluorescence units per 1.5 million cells for qPCR or as the pg per μg of total protein for the ELISAs. One-way analysis of variance (ANOVA) was preceded by the Levene test of homogeneity of variances and was used to determine overall significance. Differences between control and experimental groups were assessed with a post hoc Newman-Keuls test. Significant differences were indicated as follows: **p < 0.01, and ***p < 0.001 (versus control cultures) and ^#^*p* < 0.05, ^##^*p* < 0.01, and ^###^*p* < 0.001 (versus the cultures exposed to Aβ). The results are expressed as the mean ± SEM of 3 independent experiments. The number of replicates ranged from 5 to 12.

## Results

### PaPE-1 Inhibited Aβ-Induced LDH Release and Caspase-3 Activity in 7 DIV Neocortical Cultures After 24 H of Exposure

**Fig. 1:**
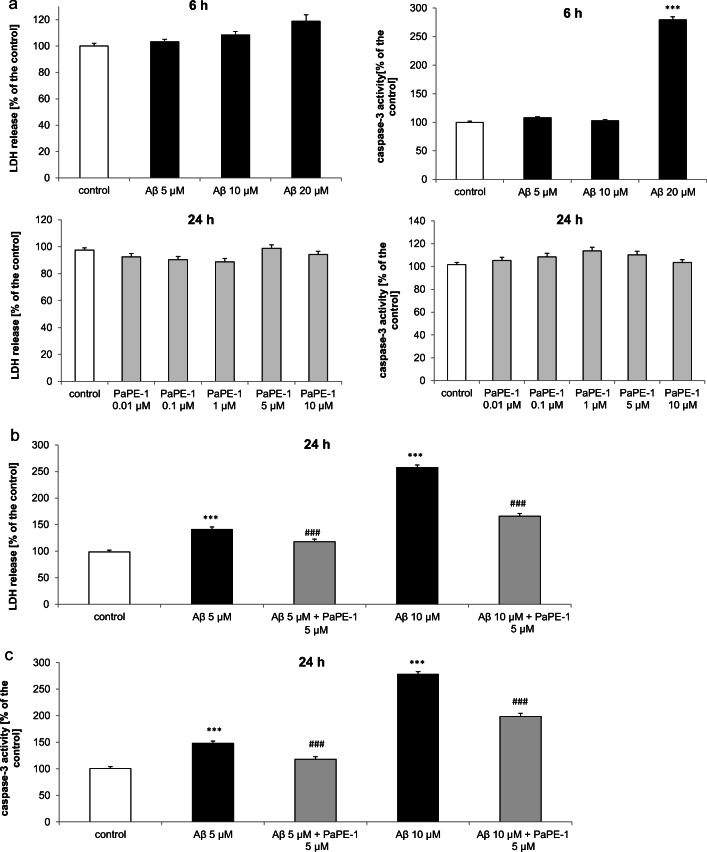
Time course and concentration response of Aβ and/or PaPE-1 on caspase-3 activity and LDH release in primary cultures of mouse neocortical cells at 7 DIV. Six hour exposure to 5-10 μM Aβ as well as 24 hour treament with PaPE-1 (0.01-10 μM) did not activate LDH or caspase-3 (panel **a**). After 24 hour treatment, 5 μM PaPE-1 inhibited the effects of Aβ in response to LDH release (panel **b**) and caspase-3 activity (panel **c**). The results are presented as a percentage of the control. Each bar represents the mean ± SEM of 3 independent experiments, consisting of 5 to 8 replicates per group. ***p < 0.001 versus the control and ^###^*p* < 0.001 versus Aβ-treated cells

In 7 DIV neocortical cultures, 24 h of exposure to Aβ (5–10 μM) induced LDH release in the range of 141–258% of the control. Cotreatment with 5 μM PaPE-1 inhibited the effects of Aβ, reaching levels of 118–166% of the control, i.e., reduced by 23–92% (Fig. 1, panel b). Moreover, Aβ (5–10 μM) increased caspase-3 activity to 148–278% of the control value. PaPE-1 (5 μM) effectively reduced Aβ-enhanced caspase-3 activity by 30–79% (Fig. 1, panel c).

Six hours of Aβ exposure to 5–10 μM Aβ did not activate LDH or caspase-3 . Following exposure to 20 μM Aβ, caspase-3 activity increased to 280% of the control. PaPE-1 (0.01–10 μM, alone) did not change LDH release or caspase-3 activity in 7 DIV neocortical cultures (Fig. 1, panel a).

### PaPE-1 Partially Reversed Aβ-Reduced Mitochondrial Membrane Potential in 7 DIV Neocortical Cultures

**Fig. 2:**
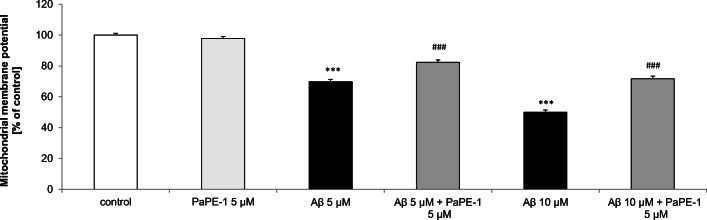
PaPE-1 partially reversed Aβ-reduced mitochondrial membrane potential in 7 DIV neocortical cultures. The results are presented as a percentage of the control. Each bar represents the mean ± SEM of 3 independent experiments, consisting of 10 replicates per group. ***p < 0.001 versus the control and ^###^*p* < 0.001 versus Aβ-treated cells

In the present study, 24 h exposure to Aβ (5–10 μM) substantially reduced the mitochondrial membrane potential to 50–70% of the control value. The effect of Aβ was partially reversed by treatment with 5 μM PaPE-1, which increased the mitochondrial membrane potential by 12–21% (Fig. 2).

Twenty-four hours of exposure to PaPE-1 (5 μM, alone) did not alter the mitochondrial membrane potential in 7 DIV neocortical cultures.

### PaPE-1 Inhibited Aβ-Increased ROS Activity in 7 DIV Neocortical Cultures

**Fig. 3:**
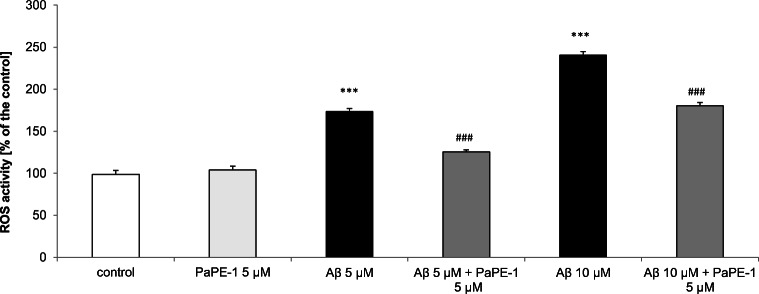
PaPE-1 inhibited Aβ-induced ROS activity in 7 DIV neocortical cultures. The results are presented as a percentage of the control. Each bar represents the mean ± SEM of 3 independent experiments, consisting of 8–12 replicates per group. ***p < 0.001 versus the control and ^###^*p* < 0.001 versus Aβ-treated cells

After 24 h of exposure to 5 and 10 μM Aβ, ROS production was enhanced and reached values of 173% and 240%, respectively. Cotreatment with 5 μM PaPE-1 inhibited the effects of Aβ, with levels 125–180% of the control, i.e., reduced by 48–60% (Fig. 3).

PaPE-1 (5 μM, alone) exposure for 24 h did not alter ROS activity in 7 DIV neocortical cultures.

### PaPE-1 Partially Reversed Aβ-Decreased Cell Viability in 7 DIV Neocortical Cultures

**Fig. 4:**
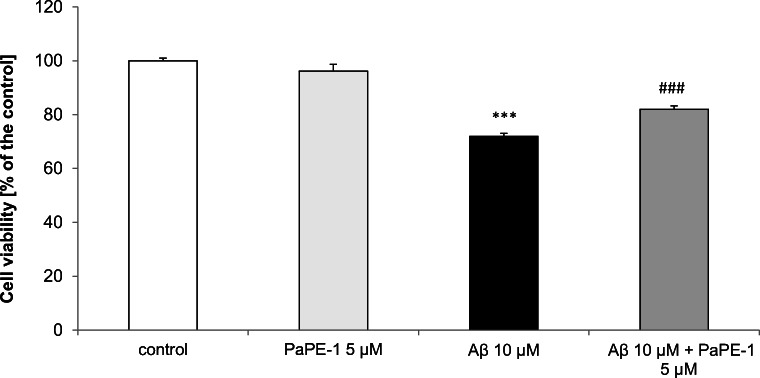
PaPE-1 (5 μM) partially reversed Aβ-decreased cell viability in 7 DIV neocortical cultures. The results are presented as a percentage of the control. Each bar represents the mean ± SEM of 3 independent experiments, consisting of 8–12 replicates per group. ***p < 0.001 versus the control and ^###^*p* < 0.001 versus Aβ-treated cells

Cell viability decreased by 28% following 24 h of exposure to 10 μM Aβ compared with control values. After treatment with 5 μM PaPE-1, the Aβ-induced decrease in cell viability was partially reversed by 10%. However, PaPE-1 alone (5 μM) did not significantly affect neuronal viability (Fig. 4).

### PaPE-1 Affected the Aβ-Increased mRNA Expression Levels of Apoptosis-Related Genes

**Fig. 5:**
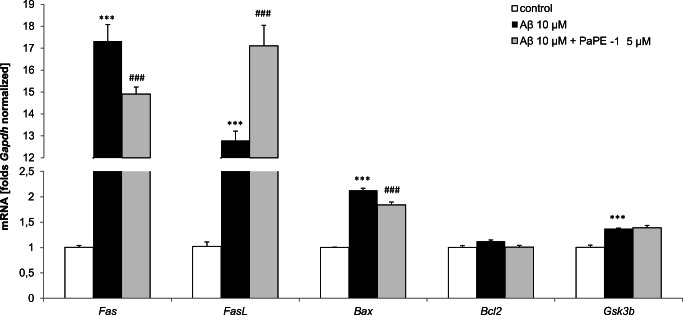
PaPE-1 affected Aβ-increased mRNA expression levels of apoptosis-related genes in neocortical cultures at 7 DIV. Cells were treated with Aβ (10 μM) or Aβ (10 μM) + PaPE-1 (5 μM) for 24 h. Each bar represents the mean ± SEM of 3 independent experiments, consisting of 6 replicates per group. ***p < 0.001 versus the control cultures, and ^###^*p* < 0.001 versus Aβ-treated cells

After 24 h of treatment with 10 μM Aβ, the mRNA expression levels of apoptosis-related genes, i.e., *Fas* (16.32-fold increase), *FasL* (11.78-fold increase), *Bax* (1.13-fold increase), and *Gsk3b* (0.37-fold increase), were increased, though expression of *Bcl2* was unaffected. Cotreatment with PaPE-1 (5 μM) inhibited Aβ-induced mRNA expression of *Fas* and *Bax* to 13.90-fold and 0.84-fold, respectively, compared with the control cells, whereas it stimulated expression of *FasL* to 16.11-fold. PaPE-1 did not influence expression of *Bcl2* or *Gsk3b* (Fig. 5).

### PaPE-1 Changed the Aβ-Increased Apoptosis-Related Protein Expression Levels

**Fig. 6:**
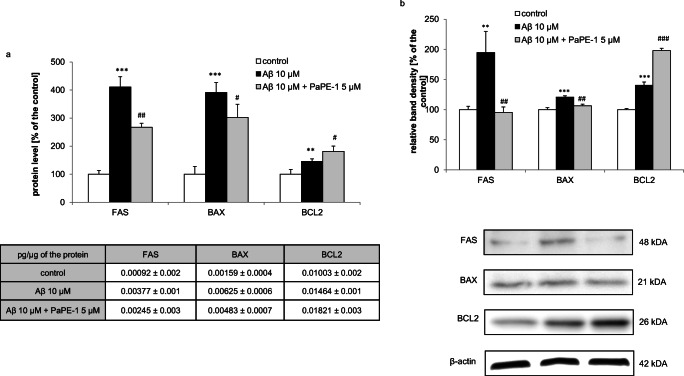
PaPE-1 changed the Aβ-induced increase in apoptosis-related protein expression levels in mouse neocortical cells. Levels of the receptors were measured using specific ELISAs (panel **a**) and western blot (panel **b**) after 24 h of treatment. Each result is presented as a percentage of the control or in terms of pg of FAS, BAX, and BCL2 per μg of total protein. Each bar represents the mean ± SEM of 3 independent experiments, consisting of 6 replicates per group. **p < 0.01 and ***p < 0.001 versus control cultures, ^#^*p* < 0.05, ^##^*p* < 0.01, and ^###^*p* < 0.001 versus Aβ-treated cells

Changes in protein levels were observed in mouse neocortical cells at 24 h after treatment. The protein levels of FAS, BAX, and BCL2 in control neocortical cultures reached 0.00092, 0.00159, and 0.01003 pg per μg of total protein, respectively, and treatment with 10 μM Aβ for 24 h increased these levels to 46–310% of the control values. Cotreatment with PaPE-1 (5 μM) decreased protein expression of FAS and BAX by 89–153% of the Aβ-induced values but increased the level of BCL2 protein expression to 182% of the control (Fig. 6, panel a). The western blot results are similar to the ELISAs. It has been observed that the treatment with 10 μM Aβ for 24 h increased protein levels of FAS, BAX, and BCL2 to 121–195% of the control values. After the cotreatment with PaPE-1 (5 μM), the protein expression of FAS and BAX decreased by 15–100% of the Aβ-induced values but the level of BCL2 protein expression increased to 198% (Fig. 6, panel b).

## Discussion

This study demonstrates for the first time that PaPE-1, which has been designed to selectively activate non-nuclear ERs, has anti-AD capacity, as evidenced in a cellular model of the disease. Following 24 h of exposure, PaPE-1 inhibited Aβ-evoked effects, as shown by reduced parameters of neurotoxicity and apoptosis, including suppressed expression of *Fas*/FAS and *Bax*/BAX and increased expression of the antiapoptotic protein BCL2. Intriguingly, we observed Aβ-stimulated expression of BCL2, which is in line with human studies showing elevated amounts of the protein in the brains of AD patients (Satou et al. 1995; O’Barr et al. 1996; Kitamura et al. 1998). Moreover, upregulation of BCL2 has been observed in APP transgenic mice, restricted to amyloid-containing brain regions (Karlnoski et al. 2007). However, PaPE-1 did not affect Aβ-stimulated expression of *Gsk3b* mRNA in our model, which suggests no interference of GSK3β-mediated apoptosis or tau hyperphosphorylation. PaPE-1 is a selective non-nuclear ER activator that does not activate classic nuclear ERs acting as transcription factors and targets only non-nuclear ERs acting via second messengers. This property positions PaPE-1 as a unique pharmacological tool that possesses the neuroprotective potential of estrogen with limited uterotrophic effects and cancer risks. Recently, PaPE-1 was shown to decrease stroke severity in a mouse model of tMCAO (Selvaraj et al. 2018), in addition to exerting cardiometabolic benefits (Gourdy et al. 2018). Therefore, our present study widens the window of pharmacological utility of PaPE-1 for the treatment of AD.

There is no relevant study comparing the effects of PaPE-1, though the model of AD is widely recognized and valuable. We showed that in mouse neocortical neurons, 24 h of treatment with Aβ (5 and 10 μM) induced apoptosis (loss of mitochondrial membrane potential, activation of caspase-3, induction of apoptosis-related genes and proteins) accompanied by increased levels of ROS and LDH as well as a reduced number of viable cells. Similar effects of Aβ were observed by Su et al. (2003), who noted upregulated expression of Fas and FasL in primary neurons, and Marquardt et al. (2017), who showed caspase-dependent apoptosis in mouse hippocampal HT22 cells following treatment with Aβ 1-42. Previous established cellular models of AD include mammalian neurons in primary cultures, human neuroblastoma SH-SY5Y and SK-N-MC cells, and teratocarcinoma NT2 cells. These cells, when treated with Aβ peptide or toxic Aβ oligomers or transfected with mtDNA from AD patients, exhibit senile plaque formation, elevated ROS production, and/or cell death. Furthermore, SH-SY5Y cells with inhibited complex I and transfected with an additional copy of the human AβPP gene show slightly elevated Aβ levels, moderately decreased ATP levels, impaired mitochondrial membrane potential, and decreased mitochondrial respiration (Stockburger et al. 2014).

According to our study, preferential activation of ER extranuclear pathways with PaPE-1 inhibits Aβ-induced apoptosis in terms of caspase-3 activity, mitochondrial membrane potential, and expression of apoptotic genes and proteins belonging to the internal (mitochondrial) or external (FAS-dependent) apoptotic pathways. Furthermore, PaPE-1 inhibits Aβ-induced ROS formation, which might in turn inhibit apoptosis, as well as prevent neuronal cell death in terms of LDH release from dead cells and CellTiter-Blue staining of viable cells. Because PaPE-1 downregulated Aβ-induced *Fas*/FAS expression but upregulated Aβ-induced *FasL* mRNA, the role of PaPE-1 in controlling the external apoptotic pathway is controversial. Nonetheless, PaPE-1 normalized the Aβ-induced loss of mitochondrial membrane potential and restored the BAX/BCL2 ratio, which suggests that the anti-AD capacity of PaPE-1 particularly relies on inhibition of the mitochondrial apoptotic pathway.

## Conclusion

In summary, our study is the first to provide evidence that preferential activation of ER extranuclear pathways with PaPE-1 protects brain neurons against Aβ-induced toxicity and that the mechanism involves inhibition of oxidative stress and apoptosis, with particular modulation of the internal/mitochondrial pathway.

## Abbreviations

AD: Alzheimer’s disease
Aβ: amyloid beta
DIV: day in vitro
ERs: estrogen receptors
ERα/ESR1: estrogen receptor alpha
ERβ/ESR2: estrogen receptor beta
GPR30/GPER1: G protein-coupled receptor 30
Gq-mER: Gq-coupled membrane estrogen receptor
LDH: lactate dehydrogenase
mERα: membrane estrogen receptor alpha
mERβ: membrane estrogen receptor beta
ROS: reactive oxygen species
tMCAO: transient middle cerebral artery occlusion
